# Consensus statements on the clinical understanding and use of bupropion in Hong Kong

**DOI:** 10.1111/cns.13376

**Published:** 2021-02-08

**Authors:** Wing‐King Lee, Kwok‐Leung Au Yeung, Ho‐Bun Lam, Chi‐Keung Wong, Ting‐Chi Wong, Chi‐Kin Fu, Shiu‐Kow Sham, Ming‐Kai Au, Tat‐Chung Lam, Daniel Ki‐Yan Mak

**Affiliations:** ^1^ Department of Psychiatry Kwai Chung Hospital Hong Kong China; ^2^ Private Practice Hong Kong China; ^3^ Department of Psychiatry Shatin Hospital Hong Kong China; ^4^ Department of Psychiatry Pamela Youde Nethersole Eastern Hospital Hong Kong China; ^5^ Private Practice Education, Prevention and Publication Subcommittee The Mental Health Association of Hong Kong Hong Kong China; ^6^ Private Practice The University of Hong Kong Hong Kong China; ^7^ Private Practice The Mental Health Association of Hong Kong Hong Kong China

**Keywords:** bupropion, consensus, Delphi technique, Hong Kong, major depressive disorder

## Abstract

**Objectives:**

To develop a local consensus to guide medical practitioners and psychiatrists on the use of bupropion in different psychiatric conditions in Hong Kong.

**Methods:**

By utilizing the modified Delphi technique, a group of 10 local physicians with extensive experience in the management of major depressive disorder (MDD) developed and voted (using an anonymous, electronic voting system) on the practicality of recommendation of a set of consensus statements on the clinical use and understanding of bupropion in Hong Kong.

**Results:**

There was a very high degree of agreement among the panelists on the 11 finalized consensus statements.

**Conclusions:**

The present consensus statements are developed as general recommendations for medical practitioners and psychiatrists to be practically referred to in clinical settings.

## 1 INTRODUCTION

Bupropion hydrochloride is a unique aminoketone antidepressant, unrelated to other known antidepressants.^1^, ^2^, ^3^ It is the only norepinephrine and dopamine reuptake inhibitor (NDRI) currently available in Hong Kong. In view of the variations in drug response due to genetic differences and the lack of local evidence‐based treatment guidelines, a consensus meeting was organized to develop guidance for psychiatrists and medical practitioners on the use of bupropion in different psychiatric conditions.

## 2 METHODS

A meeting was held on November 10, 2014, in Hong Kong, and the consensus group included 10 local physicians with extensive experience in the management of major depressive disorder (MDD). The literature search was performed in the PUBMED database using combinations of the following keywords: “bupropion,” “major depressive disorder,” “smoking cessation,” and “side effects.” After review, 32 out of 283 references were included to be discussed by the consensus group.

The consensus group utilized the modified Delphi technique to allow formal face‐to‐face expert focus meeting.^4^ After a comprehensive review and discussion, eleven statements on bupropion were finalized and voted anonymously using electronic voting devices. Each statement was rated according to both (a) quality of evidence and (b) practicability of recommendation in Hong Kong. A consensus statement was only accepted if ≥80% voted “A” or “B” for practicability (Table 1).

**TABLE 1:** The grading system for each consensus statement during the voting session. (Modified from the Canadian Task Force on the Periodic Health Examination.^35^)

Quality of evidence	Classification of recommendation	Practicability of recommendation
I: Evidence obtained from at least 1 randomized controlled trial.	A: There is good evidence to support the statement.	A: Accept completely.
II‐1: Evidence obtained from well‐designed control trials without randomization.	B: There is fair evidence to support the statement.	B: Accept with some reservation.
II‐2: Evidence obtained from well‐designed cohort or case‐control study.	C: There is poor evidence to support the statement, but recommendation made on other ground(s).	C: Accept with major reservation.
II‐3: Evidence obtained from comparison between time or places with or without intervention.	D: There is fair evidence to refute the statement.	D: Reject with reservation.
III: Opinion of respected authorities, based on clinical experience and expert committee.	E: There is good evidence to refute the statement.	E: Reject completely.

## 3 RESULTS

**Statement 1: Besides treatment for major depressive disorder, bupropion is also indicated for seasonal affective disorder and is particularly useful for patients with anhedonia, reduced motivation, weight concern, and sexual dysfunction.**

**Table:** 

Quality of evidence: I
Practicability of recommendation: A‐100%, B‐0%, C‐0%, D‐0%, E‐0%

In Hong Kong, bupropion is indicated for the treatment of MDD and seasonal affective disorder.^5^ The efficacy and safety profile of bupropion have been demonstrated in several studies.^1^, ^2^, ^6^, ^7^, ^8^ Bupropion resulted in similar effectiveness as compared to sertraline, although the side effects were significantly more common in sertraline‐treated patients.^6^ In particular, orgasm or sexual dysfunction was less common in bupropion‐treated patients.^1^, ^8^ Sexual dysfunction was reported as an adverse event by <1% of the patients,^1^ suggesting that bupropion may be the antidepressant of choice in regard to the avoidance of sexual dysfunction.

Bupropion provides greater relief to anhedonia, fatigue, and low energy, which are the most common symptoms associated with MDD patients, occurring in at least 70% of the patients.^9^, ^10^ As bupropion is also associated with weight loss,^1^, ^2^ it will also be particularly useful for patients with weight concerns.

**Statement 2: Off‐label use of bupropion includes smoking cessation, attention deficit hyperactivity disorder (ADHD), bipolar depression, depression in Parkinson's disease and occasionally for some anxiety disorders.**

**Table:** 

Quality of evidence: I (III for depression in Parkinson's disease)
Practicability of recommendation: A‐83.3%, B‐16.7%, C‐0%, D‐0%, E‐0%

The use of bupropion as an aid in smoking cessation was supported by high‐quality evidence.^3^, ^11^, ^12^ It has been shown that bupropion is effective and well tolerated in adult smokers, both healthy and medically ill with cardiovascular disease or chronic obstructive pulmonary disease (COPD).^3^ In a longer study, bupropion has also been shown to be able to delay relapse to smoking across 1 year.^3^

Patients suffering from bipolar depression can benefit from bupropion, with a low rate of manic switch, and bupropion can also be useful to relieve adult ADHD symptoms.^3^ Accumulating evidence has also shown that bupropion is efficacious in reducing depression‐related anxiety symptoms. However, the effect has not been extensively investigated.^3^

Patients with depression in Parkinson's disease could also benefit from the use of bupropion, although no randomized controlled trial has been conducted.^13^, ^14^, ^15^ Because depression in Parkinson's disease has been linked to cerebral dopaminergic hypoactivity, commonly prescribed antidepressant treatments such as tricyclic antidepressants (TCA), selective serotonin reuptake inhibitor (SSRI), and serotonin‐norepinephrine reuptake inhibitor (SNRI), which lack dopaminergic activity, may not be effective.^13^ Bupropion, with its intrinsic dopaminergic action, may be a better choice in this aspect.

**Statement 3: Bupropion is a unicyclic antidepressant with dual action on dopamine and noradrenaline, but has no effect on serotonin.**

**Table:** 

Quality of evidence: II‐2
Practicability of recommendation: A‐100%, B‐0%, C‐0%, D‐0%, E‐0%

Bupropion is classified as an atypical antidepressant, where it specifically acts as a dual norepinephrine and dopamine reuptake inhibitor (NDRI) at clinically relevant doses.^16^, ^17^, ^18^ The inhibition of serotonin uptake by bupropion and its metabolites has been shown to be negligible even at the highest concentration tested.^16^

**Statement 4: Bupropion is an effective antidepressant as monotherapy, but is also an adjuvant medication in cases of incomplete response to SSRI antidepressants.**

**Table:** 

Quality of evidence: I
Practicability of recommendation: A‐100%, B‐0%, C‐0%, D‐0%, E‐0%

In case of suboptimal response to the medication, adding bupropion as an augmentation may also be considered. The augmentation of SSRI with bupropion has demonstrated benefits, including greater reduction in the number and severity of symptoms and fewer side effects.^8^

**Statement 5: Bupropion is generally well tolerated; the most common side effects include dry mouth, headache, and insomnia; it should be avoided at bedtime.**

**Table:** 

Quality of evidence: I
Practicability of recommendation: A‐100%, B‐0%, C‐0%, D‐0%, E‐0%

The safety profile of bupropion has been studied extensively in thousands of clinical trial subjects and in over 40 million patients who have received bupropion clinically.^3^, ^5^ Bupropion has been shown to be generally well tolerated and resulted in a relativity low rate of discontinuation.^3^ Insomnia is usually transient and can be avoided by not taking bupropion near or at bedtime. In addition, the consensus group acknowledged that dry mouth is relatively common in Chinese patients.

**Statement 6: Bupropion should be used with caution in patients with hypertension or cardiovascular comorbidities. It is not recommended in patients at risk of seizures, eating disorder, psychosis, and closed‐angle glaucoma.**

**Table:** 

Quality of evidence: I
Practicability of recommendation: A‐100%, B‐0%, C‐0%, D‐0%, E‐0%

Cautions should be exercised when prescribing bupropion in patients with hypertension or cardiovascular comorbidities. Although the use of bupropion as a monotherapy has not been associated with effects on blood pressure as compared to placebo in clinical trials, the risk in elevated blood pressure is increased if bupropion is used in a combination therapy with other drugs that also affect the dopaminergic and noradrenergic activity.^3^

Bupropion should not be used in patients with closed‐angle glaucoma due to the risk of angle‐closure attack.^5^ Bupropion is also not recommended in patients that have or had an eating disorder such as bulimia.^5^ Similar with SSRI, bupropion is also associated with the risk of seizure, where the rate increases substantially at doses above 450 mg/d.^3^ The group acknowledged the suggestion to exclude patients with past history of epilepsy and to screen patients for comorbidities or medications that may lower seizure threshold. Furthermore, to reduce the risk of seizure, bupropion should be started on a low dose and increased gradually to achieve therapeutic effect.

Finally, bupropion is also not recommended in patients with history of psychosis. Bupropion has a potential risk of precipitating or worsening psychosis in selected at‐risk populations, since excessive dopaminergic activity are suspected to be linked to the pathogenesis of psychosis.^19^

**Statement 7: Bupropion should not be used in patients receiving monoamine oxidase inhibitors, and its combination with venlafaxine may lead to a dose‐dependent increase in blood pressure.**

**Table:** 

Quality of evidence: I (III for combination with venlafaxine)
Practicability of recommendation: A‐100%, B‐0%, C‐0%, D‐0%, E‐0%

Monoamine oxidase inhibitors (MAOIs) should not be taken together with bupropion because there is an increased risk of hypertensive reactions. A washout period of at least 14 days is required when a patient switches between the two drugs.^5^ Moreover, bupropion and its metabolites are CYP2D6 inhibitors, whereas venlafaxine is a known substrate of CYP2D6. Hence, the concomitant use of bupropion and venlafaxine would shift the drug plasma levels toward an increased level of venlafaxine.^20^, ^21^

**Statement 8: Smoking and alcohol use do not appear to interact with bupropion, but its serum concentration may be increased by zinc supplement.**

**Table:** 

Quality of evidence: II‐2
Practicability of recommendation: A‐83.3%, B‐16.7%, C‐0%, D‐0%, E‐0%

There is no evidence for kinetic interactions between smoking or alcohol with bupropion.^12^, ^22^ Zinc supplement might produce synergistic effects when given in combination with bupropion^23^; physicians should be aware of this interaction.

**Statement 9: Bupropion overdose rarely results in death but may lead to seizures, hallucinations, delusions, vomiting, and aggressive behavior.**

**Table:** 

Quality of evidence: II‐3
Practicability of recommendation: A‐100%, B‐0%, C‐0%, D‐0%, E‐0%

Bupropion overdose, either in adults or children, seems to lack major cardiovascular toxicity to result in death; the symptoms are predominantly neurological.^24^ Accidental ingestion of bupropion in children showed limited toxicity.^25^, ^26^, ^27^, ^28^, ^29^

**Statement 10: Abrupt withdrawal of bupropion can result in discontinuation syndrome, which may manifest as dystonia, irritability, anxiety, mania, headache, aches, and pains.**

**Table:** 

Quality of evidence: III
Practicability of recommendation: A‐100%,B‐0%,C‐0%,D‐0%,E‐0%

Although withdrawal symptoms associated with most antidepressants are well known,^5^ the exact pathophysiologic explanations are not yet clear. The consensus group acknowledged this risk and advised that the discontinuation of bupropion should be performed by tapering the drug slowly and patients should be educated about the related symptoms.

**Statement 11: Use of bupropion during pregnancy may lead to fetal cardiac malformation during first trimester.**

**Table:** 

Quality of evidence: II‐3
Practicability of recommendation: A‐100%, B‐0%, C‐0%, D‐0%, E‐0%

There are contradicting study results regarding the effect of bupropion on the risk to fetal cardiac malformation during the first trimester of pregnancy.^30^, ^31^, ^32^, ^33^, ^34^ Nevertheless, the consensus group agreed that there is an elevated risk for it. A statistically significant association between bupropion and fetal cardiac malformation was found, although the magnitude of the observed risks was small.^30^, ^33^ Ideally, the obstetrician and psychiatrist should work together to assess the risk/benefit of using bupropion and counsel the patient on the potential risk for the developing fetus.^30^

## 4 CONCLUSION

The consensus group has unanimously reached an agreement on 11 statements regarding the use of bupropion in Hong Kong. The present consensus statements are developed as general recommendations for medical practitioners and psychiatrists to be practically referred to in clinical settings.

## CONFLICT OF INTEREST

The authors declare no conflict of interest.

## MISSION OF THE AANP

The AANP aims to become the recognized forum to foster ongoing local and international collaboration, on education intended to advance the treatment of all aspects of mood disorder and psychotic illnesses, particularly schizophrenia, and to improve the outcomes and quality of life for those who suffer from psychiatric illness and for their carers and family members.
